# *trans*-Diamminebis(1,2-di­cyano­ethene-1,2-di­thiol­ato)platinum(IV)

**DOI:** 10.1107/S2414314620009803

**Published:** 2020-07-28

**Authors:** Mahaa J. Siddiqui, Volodymyr V. Nesterov, Matthew T. Steidle, Bradley W. Smucker

**Affiliations:** a Austin College, 900 N Grand, Sherman, TX 75090, USA; b Department of Chemistry, University of North Texas, 1508 W. Mulberry, Denton, TX, 76201, USA; Howard University, USA

**Keywords:** crystal structure, platinum(IV), di­thiol­ene

## Abstract

The title compound, [Pt(C_4_N_2_S_2_)_2_(NH_3_)_2_], represents an octa­hedral platinum(IV) complex with two *trans-*ammine and two mnt (mnt = 1,2-di­cyano­ethene-1,2-di­thiol­ato) ligands. The Pt—N and Pt—S distances are consistent with those in other platinum(IV) complexes. As a result of a slight canting of the coordination of the mnt ligand to the platinum(IV) atom, the nitrile nitro­gen atoms are positioned suitably to hydrogen-bond with adjacent ammines.

## Abstract 2

The structure of the *trans*-Pt(NH_3_)_2_(mnt)_2_ complex has Pt—N and Pt—S distances that are are consistent with those in other platinum(IV) complexes. The nitrile nitro­gen atoms are positioned suitably to hydrogen bond with adjacent ammines.

## Graphical abstract

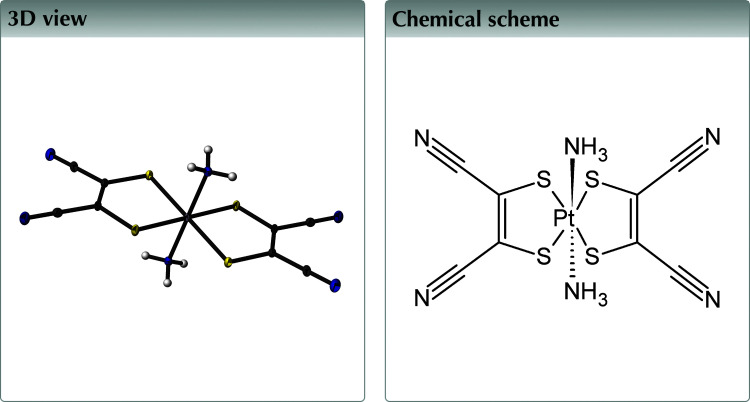

## Structure description

**Figure 1:**
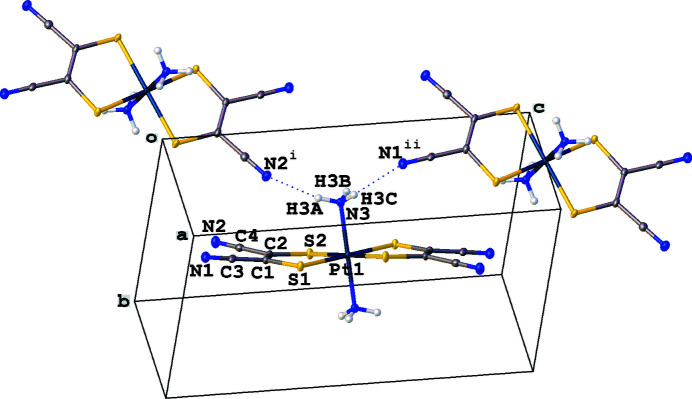
Displacement ellipsoid plot 50% probability of all non-H atoms showing N—H hydrogen bonding between nitrile nitro­gen atoms and hydrogen atoms on adjacent ammines. Symmetry codes: (i) −*x*, *y* − 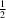 , −*z* + 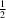 ; (ii) *x*, −*y* + 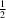 , *z* + 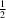 .

The neutral title complex contains two ammine and two mnt ligands forming an octa­hedral platinum(IV) complex. The C—S distances of 1.747 (3) and 1.744 (3) Å and the C=C distance of 1.358 (4) Å support the ene-1,2-di­thiol­ate form of the mnt ligand (Güntner *et al.*, 1989 ▸; Chandrasekaran *et al.*, 2014 ▸). The two ammine ligands are *trans* with a Pt—N bond length of 2.055 (2) Å, which is consistent with the Pt—N distances in other platinum(IV) complexes of 2.056 (9) (Fanwick & Huckaby, 1982 ▸) and 2.053 (5) Å (Brawner *et al.*, 1978 ▸). The Pt—S distances of 2.3434 (8) and 2.3461 (7) Å are longer than in square-planar platinum complexes with mnt such as the Pt^II^—S distances of 2.290 and 2.282 Å in [Pt(mnt)_2_]^2−^ (Günter *et al.*, 1989 ▸) or the Pt^III^—S distance of 2.262 Å in [Pt(mnt)_2_]^−^ (Mochida *et al.*, 2010 ▸). This longer Pt—S bond is comparable, however, with the Pt—S distance of 2.3619 Å found in a similar octa­hedral platinum(IV) complex with two di­thiol­ene and two *trans* phosphine ligands (Chandrasekaran *et al.*, 2014 ▸). The coordination of the mnt ligands is slightly canted from the platinum(IV) atom, which allows for hydrogen bonding between the nitrile nitro­gen atoms and adjacent ammines (Fig. 1 ▸, Table 1 ▸). These interactions lead to the formation of a three-dimensional network.

## Synthesis and crystallization

A solution of 13.9 mg (7.46 × 10 ^−5^mol) of Na_2_mnt dissolved in 10 mL of water was combined with a solution of 25 mg (7.48 × 10^−5^ mol) of tetra­ammineplatinum(II) chloride dissolved in 25 mL of water, and stirred for 2 h in air. The solvent was removed using a vacuum oven to give 26.5 mg of a brown product isolated [^1^H NMR (*d*-DMSO) 4.23ppm]. Light-orange crystals of the title compound were grown by liquid diffusion of diethyl ether into a methanol solution of the synthesized product in a tall, narrow tube that was covered with parafilm. The platinum(II) di­thiol­ene complex is presumed to oxidize to the ammine-stabilized octa­hedral platinum(IV) di­thiol­ene compound *via* air, demonstrating a synthetic route toward stable neutral Pt^IV^ di­thiol­ene complexes (Geiger *et al.*, 2001 ▸).

## Refinement

Crystal data, data collection and structure refinement details are summarized in Table 2 ▸.

## Supplementary Material

Crystal structure: contains datablock(s) I. DOI: 10.1107/S2414314620009803/bv4032sup1.cif

Structure factors: contains datablock(s) I. DOI: 10.1107/S2414314620009803/bv4032Isup2.hkl

CCDC reference: 2017151

Additional supporting information:  crystallographic information; 3D view; checkCIF report

## Figures and Tables

**Table 1:** Hydrogen-bond geometry (Å, °)

*D*—H⋯*A*	*D*—H	H⋯*A*	*D*⋯*A*	*D*—H⋯*A*
N3—H3*A*⋯N2^i^	0.89	2.16	3.016 (3)	160
N3—H3*B*⋯S2^ii^	0.89	2.73	3.610 (3)	171
N3—H3*C*⋯N1^iii^	0.89	2.26	3.011 (3)	142

Symmetry codes: (i) 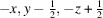 ; (ii) 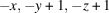 ; (iii) 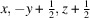 .

**Table 2:** Experimental details

Crystal data	
Chemical formula	[Pt(C_4_N_2_S_2_)_2_(NH_3_)_2_]
*M*_r_	509.52
Crystal system, space group	Monoclinic, *P*2_1_/*c*
Temperature (K)	100
*a*, *b*, *c* (Å)	6.1778 (3), 7.7700 (4), 14.8862 (7)
β (°)	95.935 (4)
*V* (Å^3^)	710.73 (6)
*Z*	2
Radiation type	Mo *K*α
μ (mm^−1^)	10.45
Crystal size (mm)	0.05 × 0.02 × 0.01

Data collection	
Diffractometer	Rigaku XtaLAB Synergy, Dualflex, HyPix
Absorption correction	Multi-scan (*CrysAlis PRO*; Rigaku OD, 2019 ▸)
*T*_min_, *T*_max_	0.509, 1.000
No. of measured, independent and observed [*I* > 2σ(*I*)] reflections	8027, 1556, 1375
*R*_int_	0.034
(sin θ/λ)_max_ (Å^−1^)	0.641

Refinement	
*R*[*F* ^2^ > 2σ(*F* ^2^)], *wR*(*F* ^2^), *S*	0.017, 0.038, 1.05
No. of reflections	1556
No. of parameters	89
H-atom treatment	H-atom parameters constrained
Δρ_max_, Δρ_min_ (e Å^−3^)	0.62, −0.54

Computer programs: *CrysAlis PRO* (Rigaku OD, 2019 ▸), *SHELXT* (Sheldrick, 2015*a* ▸), *SHELXL* (Sheldrick, 2015*b* ▸) and *OLEX2* (Dolomanov *et al.*, 2009 ▸).

## full crystallographic data

### Crystal data

**Table:**

[Pt(C_4_N_2_S_2_)_2_(NH_3_)_2_]	*F*(000) = 476
*M**_r_* = 509.52	*D*_x_ = 2.381 Mg m^−^^3^
Monoclinic, *P*2_1_/*c*	Mo *K*α radiation, λ = 0.71073 Å
*a* = 6.1778 (3) Å	Cell parameters from 5207 reflections
*b* = 7.7700 (4) Å	θ = 2.7–29.7°
*c* = 14.8862 (7) Å	µ = 10.45 mm^−^^1^
β = 95.935 (4)°	*T* = 100 K
*V* = 710.73 (6) Å^3^	Plate, clear light orange
*Z* = 2	0.05 × 0.02 × 0.01 mm

### Data collection

**Table:**

Rigaku XtaLAB Synergy, Dualflex, HyPix diffractometer	1556 independent reflections
Radiation source: micro-focus sealed X-ray tube, PhotonJet (Mo) X-ray Source	1375 reflections with *I* > 2σ(*I*)
Mirror monochromator	*R*_int_ = 0.034
Detector resolution: 10.0000 pixels mm^-1^	θ_max_ = 27.1°, θ_min_ = 2.8°
ω scans	*h* = −7→7
Absorption correction: multi-scan (CrysAlisPro; Rigaku OD, 2019)	*k* = −9→9
*T*_min_ = 0.509, *T*_max_ = 1.000	*l* = −18→19
8027 measured reflections	

### Refinement

**Table:**

Refinement on *F*^2^	Primary atom site location: dual
Least-squares matrix: full	Hydrogen site location: inferred from neighbouring sites
*R*[*F*^2^ > 2σ(*F*^2^)] = 0.017	H-atom parameters constrained
*wR*(*F*^2^) = 0.038	*w* = 1/[σ^2^(*F*_o_^2^) + (0.0154*P*)^2^ + 0.3678*P*] where *P* = (*F*_o_^2^ + 2*F*_c_^2^)/3
*S* = 1.05	(Δ/σ)_max_ < 0.001
1556 reflections	Δρ_max_ = 0.62 e Å^−^^3^
89 parameters	Δρ_min_ = −0.54 e Å^−^^3^
0 restraints	

### Special details

**Table:**

Geometry. All esds (except the esd in the dihedral angle between two l.s. planes) are estimated using the full covariance matrix. The cell esds are taken into account individually in the estimation of esds in distances, angles and torsion angles; correlations between esds in cell parameters are only used when they are defined by crystal symmetry. An approximate (isotropic) treatment of cell esds is used for estimating esds involving l.s. planes.

### Fractional atomic coordinates and isotropic or equivalent isotropic displacement parameters (Å^2^)

**Table:**

	*x*	*y*	*z*	*U*_iso_*/*U*_eq_	
Pt1	0.500000	0.500000	0.500000	0.00819 (6)	
S1	0.62740 (13)	0.46855 (9)	0.35800 (5)	0.01208 (16)	
S2	0.20123 (12)	0.65846 (10)	0.43437 (5)	0.01294 (16)	
N1	0.4195 (5)	0.4964 (3)	0.1195 (2)	0.0179 (6)	
N2	−0.0905 (4)	0.7217 (3)	0.20727 (17)	0.0182 (6)	
N3	0.3182 (4)	0.2794 (3)	0.47944 (16)	0.0125 (5)	
H3A	0.283811	0.263528	0.420476	0.015*	
H3B	0.197076	0.289137	0.506596	0.015*	
H3C	0.395109	0.189825	0.502333	0.015*	
C1	0.4056 (5)	0.5506 (4)	0.2889 (2)	0.0110 (6)	
C2	0.2301 (5)	0.6263 (4)	0.32026 (18)	0.0116 (6)	
C3	0.4129 (5)	0.5226 (3)	0.1948 (2)	0.0120 (6)	
C4	0.0516 (5)	0.6804 (4)	0.25742 (19)	0.0124 (6)	

### Atomic displacement parameters (Å^2^)

**Table:**

	*U*^11^	*U*^22^	*U*^33^	*U*^12^	*U*^13^	*U*^23^
Pt1	0.00671 (9)	0.01158 (10)	0.00598 (9)	0.00083 (6)	−0.00073 (6)	0.00050 (6)
S1	0.0099 (4)	0.0183 (4)	0.0079 (3)	0.0023 (3)	0.0005 (3)	0.0006 (3)
S2	0.0104 (3)	0.0193 (4)	0.0088 (3)	0.0045 (3)	−0.0006 (3)	0.0021 (3)
N1	0.0231 (16)	0.0163 (15)	0.0136 (14)	0.0000 (11)	−0.0009 (12)	0.0000 (10)
N2	0.0171 (14)	0.0228 (15)	0.0143 (13)	0.0030 (12)	−0.0008 (11)	0.0019 (11)
N3	0.0099 (13)	0.0157 (13)	0.0117 (12)	0.0002 (10)	−0.0008 (10)	0.0027 (10)
C1	0.0123 (15)	0.0119 (14)	0.0085 (14)	−0.0017 (12)	−0.0010 (12)	0.0009 (11)
C2	0.0136 (15)	0.0119 (15)	0.0086 (14)	−0.0020 (12)	−0.0028 (11)	0.0024 (11)
C3	0.0149 (16)	0.0076 (15)	0.0124 (16)	0.0001 (11)	−0.0035 (12)	0.0008 (11)
C4	0.0135 (15)	0.0111 (15)	0.0127 (14)	−0.0019 (12)	0.0014 (12)	−0.0002 (12)

### Geometric parameters (Å, º)

**Table:**

Pt1—S1^i^	2.3434 (8)	N1—C3	1.143 (4)
Pt1—S1	2.3434 (8)	N2—C4	1.138 (4)
Pt1—S2	2.3461 (7)	N3—H3A	0.8900
Pt1—S2^i^	2.3461 (7)	N3—H3B	0.8900
Pt1—N3^i^	2.055 (2)	N3—H3C	0.8900
Pt1—N3	2.055 (2)	C1—C2	1.358 (4)
S1—C1	1.747 (3)	C1—C3	1.423 (4)
S2—C2	1.744 (3)	C2—C4	1.433 (4)
			
S1^i^—Pt1—S1	180.0	C2—S2—Pt1	100.09 (10)
S1^i^—Pt1—S2	89.87 (3)	Pt1—N3—H3A	109.5
S1—Pt1—S2^i^	89.87 (3)	Pt1—N3—H3B	109.5
S1—Pt1—S2	90.13 (3)	Pt1—N3—H3C	109.5
S1^i^—Pt1—S2^i^	90.13 (3)	H3A—N3—H3B	109.5
S2^i^—Pt1—S2	180.0	H3A—N3—H3C	109.5
N3^i^—Pt1—S1^i^	90.46 (7)	H3B—N3—H3C	109.5
N3—Pt1—S1^i^	89.54 (7)	C2—C1—S1	124.1 (2)
N3—Pt1—S1	90.46 (7)	C2—C1—C3	120.8 (3)
N3^i^—Pt1—S1	89.54 (7)	C3—C1—S1	114.9 (2)
N3^i^—Pt1—S2	91.06 (7)	C1—C2—S2	124.2 (2)
N3—Pt1—S2^i^	91.06 (7)	C1—C2—C4	119.4 (3)
N3—Pt1—S2	88.94 (7)	C4—C2—S2	116.4 (2)
N3^i^—Pt1—S2^i^	88.94 (7)	N1—C3—C1	178.5 (3)
N3—Pt1—N3^i^	180.0	N2—C4—C2	179.3 (3)
C1—S1—Pt1	100.20 (11)		
			
Pt1—S1—C1—C2	6.6 (3)	S1—C1—C2—S2	1.7 (4)
Pt1—S1—C1—C3	−169.3 (2)	S1—C1—C2—C4	−176.5 (2)
Pt1—S2—C2—C1	−8.8 (3)	C3—C1—C2—S2	177.4 (2)
Pt1—S2—C2—C4	169.4 (2)	C3—C1—C2—C4	−0.8 (5)

Symmetry code: (i) −*x*+1, −*y*+1, −*z*+1.

### Hydrogen-bond geometry (Å, º)

**Table:**

*D*—H···*A*	*D*—H	H···*A*	*D*···*A*	*D*—H···*A*
N3—H3*A*···N2^ii^	0.89	2.16	3.016 (3)	160
N3—H3*B*···S2^iii^	0.89	2.73	3.610 (3)	171
N3—H3*C*···N1^iv^	0.89	2.26	3.011 (3)	142

Symmetry codes: (ii) −*x*, *y*−1/2, −*z*+1/2; (iii) −*x*, −*y*+1, −*z*+1; (iv) *x*, −*y*+1/2, *z*+1/2.
